# Renal sympathetic denervation guided by renal nerve stimulation to treat ventricular arrhythmia in CKD patients with ICD

**DOI:** 10.18632/oncotarget.16278

**Published:** 2017-03-16

**Authors:** Márcio Galindo Kiuchi, Shaojie Chen, Luis Marcelo Rodrigues Paz, Helmut Pürerfellner

**Affiliations:** ^1^ Department of Medicine, Division of Artificial Cardiac Stimulation, Hospital e Clínica São Gonçalo, São Gonçalo, Rio de Janeiro, Brazil; ^2^ Department of Cardiology, Shanghai First People's Hospital, Shanghai Jiao Tong University School of Medicine, Shanghai, China; ^3^ Department of Cardiology, Elisabethinen University Teaching Hospital Linz, Linz, Austria

**Keywords:** ventricular arrhythmias, anti-tachycardia therapy pacing, automatic cardioverter-defibrillator, chronic renal disease, renal sympathetic denervation

## Abstract

Chronic kidney disease (CKD) patients on stage 4 present greater risk rates for malignant ventricular arrhythmia events. This study examined patients with CKD in stages 1, 2, 3 and 4, left ventricular dysfunction and automatic implantable cardioverter-defibrillator (ICD). Our goal was to record the appropriate therapies, “Anti-tachycardia Therapy Pacing” (ATP) and shock events during the 18 months of follow-up and compare the incidence and severity of these at different stages of CKD, mainly in patients with CKD stage 4 underwent renal sympathetic denervation (RSD) guided by renal nerve stimulation (RNS). One hundred and fifteen patients were evaluated once every three months till 18 months of follow-up. The arrhythmic events were assessed at each follow-up visit. Comparing the groups, we can see the number of ATP and shock events recorded by ICD during 18 months of follow-up, and differences in the number of therapeutic events between the various stages of CKD. The hazard ratio (HR), 95% confidence interval (CI) and P value for ATP and shock events between all the CKD stages were evaluated by the log-rank/Mantel-Haenszel test. At the 18^th^ month of follow-up, 75% of patients with CKD stage 4 received ATP, and 70% were treated with shock while only 20% of the subjects with CKD stage 4 that were submitted to RSD received ATP and 20% were treated with shock, P<0.0001 and P=0.0002, respectively. In our study, a decline occurred in the incidence of arrhythmias, and therefore, appropriate ICD therapies in advanced stages of CKD, reducing the risk rates for these events in patients with CKD on stage 4 after RSD guided by RNS in comparison to the other CKD stages. Our results suggest that RSD can control the higher incidence of malignant arrhythmias in advanced CKD stages.

## INTRODUCTION

Sudden cardiac death (SCD) represents almost 1/3 of the 1,000.000/year of deaths due to cardiovascular illness according to U.S. statistics [1, 2]. The significance of automatic implantable cardioverter-defibrillator (ICD) has been confirmed in patients with prior myocardial infarction and grave systolic left ventricular dysfunction for secondary prevention [3–5]. The remaining from heart arrest or those ones with nonstop ventricular tachycardia presents a great possibility of a repetition of such events [6]. The strategies to treat this condition comprise the usage of antiarrhythmic medications, resection by cardiac surgery, percutaneous ablation of the epicardium or/and endocardium, and the implant of ICDs.

The excessive activity of the sympathetic nervous system has keyrole with regard to increased cardiovascular danger in individuals who have renal impairment [7–9]. In the chronic kidney disease (CKD), sympathetic high activity is apparent at the initial stages, presenting a close connection with the gravity of the end stage of renal disease [5, 10–13]. Such as the reduction in the glomerular filtration rate arises, there is correspondingly an upsurge in cardiovascular occurrences and death in CKD subjects [5, 14], particularly due to arrhythmias and their aftereffects. Our group earlier described that in the occurrence of ventricular tachycardia, the anti-tachycardia pacing therapy (ATP) or synchronized cardioversion shock, and in cases of ventricular fibrillation detection, the ICD releases an unsynchronized shock of great energy defibrillation. As observed, these kinds of occurrences are Happen more in individuals with CKD on stage 4 [5, 15].

The present study evaluated CKD subjects on stages 1, 2, 3 and 4 who underwent ICD implant, for a period up to 18 months of follow-up. We aim to compare the incidence and severity of the ATP and shock therapies during the 18 months of follow-up at these different stages of CKD, mainly in patients with CKD stage 4 submitted to renal sympathetic denervation (RSD) guided by previous renal nerve stimulation (RNS) [5].

## RESULTS

### Patients

The 115 individuals who had all the inclusion criteria were comprised in the assessment [5]. The starting point features divided by CKD stages into five groups, are displayed meticulously in Table 1.

**Table 1:** Baseline features

	CKD stage 1	CKD stage 2	CKD stage 3	CKD stage 4	CKD stage 4+RSD	Overall P value
N	25	25	25	20	20	---
Age, years	60.0±11.5	66.2±13.4	69.1±16.0	64.0±15.5	70.0±13.0	0.1024
Body mass index, kg/m^2^	28.1±6.8	29.0±5.0	27.4±5.5	27.3±6.3	26.8±6.8	0.7691
Male gender (%)	20 (80%)	19 (76%)	17 (68%)	16 (80%)	13 (65%)	0.6993
White ethnicity (%)	17 (68%)	20 (80%)	16 (64%)	15 (75%)	16 (80%)	0.6435
Type 2 Diabetes *Mellitus* (%)	18 (72%)	17 (68%)	13 (52%)	10 (50%)	12 (60%)	0.5154
Coronary artery disease	22 (88%)	21 (84%)	21 (84%)	16 (80%)	17 (85%)	0.9685
Ischemic etiology	22 (88%)	21 (84%)	23 (92%)	16 (80%)	17 (85%)	0.8203
Antiarrhythmic agent						
Amiodarone	25 (100%)	25 (100%)	25 (100%)	20 (100%)	20 (100%)	1.0000
Antihypertensive agents						
ACEI/ARB	25 (100%)	25 (100%)	25 (100%)	20 (100%)	20 (100%)	1.0000
Spironolactone	25 (100%)	25 (100%)	25 (100%)	20 (100%)	20 (100%)	1.0000
DHP Ca^++^ channel blockers	15 (60%)	14 (56%)	15 (60%)	10 (50%)	11 (55%)	0.9615
β-blockers	25 (100%)	25 (100%)	25 (100%)	20 (100%)	20 (100%)	1.0000

The values are presented as mean ± SD or %; ACEI, receptor inhibitor of angiotensin converting enzyme; ARB, angiotensin receptor blocker; CKD, chronic kidney disease (stages 1, 2, 3 and 4); DHP, dihydropyridyne; N, number of patients; +RSD, patients that underwent renal sympathetic denervation.

### Mean 24-hour ABPM and renal function

As we can observe in Table 2, by definition, there were significant differences in creatinine levels comparing the stages of CKD consequently it reflects the differences in the eGFR, as well as, ACR at the 18^th^ month of follow-up, just for some comparisons. No significant difference was observed in the mean 24-hour ABPM, in the comparison between baseline *vs*. 18 months of follow-up for the same group or between CKD stages.

**Table 2:** Mean 24-hour ABPM and renal function during 18 months of follow-up

Baseline	CKD stage 1 (n=25)	CKD stage 2 (n=25)	CKD stage 3 (n=25)	CKD stage 4 (n=20)	CKD stage 4+RSD (n=20)	Overall P value for comparisons between groups
Creatinine, mg/dL	0.80±0.03	1.10±0.10	1.59±0.10^††^	2.62±0.08	2.61±0.10^▲†^	<0.0001 for all comparisons except for CKD stage 4 *vs*. 4+RSD
eGFR, mL/min/1.73m^2^	97.3±7.2	70.3±6.7	44.0±5.8^†^	25.0±3.2	24.0±2.6^▲††^	<0.0001 for all comparisons except for CKD stage 4 *vs*. 4+RSD
ACR, mg/g	43.2±10.7	52.3±12.5	59.0±13.1^†^	74.8±15.0^†^	77.9±12.7^††^	<0.0001^*^
Mean 24-hour ABPM, mmHg	121±6/75±3	122±6/75±4	121±6/76±4	123±7/75±4	122±8/76±2	0.8360/0.6978
12^th^ month of follow-up	CKD stage 1 (n=25)	CKD stage 2 (n=25)	CKD stage 3 (n=25)	CKD stage 4 (n=20)	CKD stage 4+RSD (n=20)	Overall P value for comparisons between groups
Creatinine, mg/dL	0.81±0.05	1.11±0.09	1.70±0.08	2.65±0.11	2.10±0.50	<0.0001 for all comparisons
eGFR, mL/min/1.73m^2^	96.7±8.0	68.0±7.3	40.3±4.6	24.3±2.9	32.7±3.5	<0.0001 for all comparisons
ACR, mg/g	45.0±9.0	55.4±13.1	68.3±17.0	88.6±13.9	50.2±12.0	<0.0001^*/*^*
Mean 24-hour ABPM, mmHg	123±7/74±3	123±7/76±5	123±8/76±4	124±7/77±3	120±8/74±6	0.4978/0.0848
18^th^ month of follow-up	CKD stage 1 (n=25)	CKD stage 2 (n=25)	CKD stage 3 (n=25)	CKD stage 4 (n=20)	CKD stage 4+RSD (n=20)	Overall P value for comparisons between groups
Creatinine, mg/dL	0.82±0.06	1.13±0.07^▲^	1.73±0.10	2.68±0.70	2.00±0.70^▲^	<0.0001 for all comparisons except for CKD stage 1 *vs*. 2, and 3 *vs*. 4+RSD
eGFR, mL/min/1.73m^2^	96.0±8.4	66.7±6.5	38.0±4.7	21.5±4.0^†^	37.8±3.3^▲††^	<0.0001 for all comparisons except for CKD stage 3 *vs*. 4+RSD
ACR, mg/g	49.6±13.0	58.3±11.6	70.2±10.0	98.1±10.0	42.1±8.8	<0.0001^*/*^*
Mean 24-hour ABPM, mmHg	120±8/74±4	121±9/76±3	120±7/77±4	123±6/77±5	118±8/75±5	0.3501/0.0663

The values are presented as mean ± SD. ABPM, ambulatory blood pressure monitoring; ACR, albumin:creatinine ratio; CKD, chronic kidney disease (stage 1, 2, 3 and 4); eGFR, estimated glomerular filtration rate; +RSD, patients that underwent renal sympathetic denervation. Comparisons between groups, Creatinine: at baseline ^▲^P=0.9960 for CKD stage 4 vs. 4+RSD; at 18^th^ month ^▲^P=0.0714 and ^▲^P=0.2021 for CKD stage 1 *vs*. 2, and for CKD stage 3 *vs*. 4+RSD, respectively; eGFR (CKD-EPI): at baseline ^▲^P=0.9799 for CKD stage 4 vs. 4+RSD; at 18^th^ month ^▲^P>0.9999 for CKD stage 3 *vs*. 4+RSD; ACR: at baseline ^*^P<0.0001 for CKD stage 1 *vs*. 3, for CKD stage 1 *vs*. 4, for CKD stage 1 *vs*. 4+RSD, for CKD stage 2 *vs*. 4, for CKD stage 2 *vs*. 4+RSD, for CKD stage 3 *vs*. 4, for CKD stage 3 *vs*. 4+RSD; at 12^th^ month *P<0.05 for CKD stage 2 *vs*. 3, and ^*^P<0.0001 for CKD stage 1 *vs*. 3, for CKD stage 1 *vs*. 4, for CKD stage 2 *vs*. 4, for CKD stage 3 *vs*. 4, for CKD stage 3 *vs*. 4+RSD, for CKD stage 4 *vs*. 4+RSD; at 18^th^ month *P<0.05 for CKD stage 1 *vs*. 2, for CKD stage 2 *vs*. 3, and ^*^P<0.0001 for CKD stage 1 *vs*. 3, for CKD stage 1 *vs*. 4, for CKD stage 2 *vs*. 4, for CKD stage 2 *vs*. 4+RSD, for CKD stage 3 *vs*. 4, for CKD stage 3 *vs*. 4+RSD, for CKD stage 4 *vs*. 4+RSD. Comparisons into the same group, ACR: ^†^P<0.05 for baseline *vs*. 12^th^ month, for baseline *vs*. 18^th^ month in CKD stage 3 and 4 groups, and ^††^P<0.0001 for baseline *vs*. 12^th^ month, for baseline *vs*. 18^th^ month in CKD stage 4+RSD; creatinine: ^††^P<0.0001 for baseline *vs*. 12^th^ month, for baseline *vs*. 18^th^ month in CKD stage 3 group, and ^†^P<0.05 for baseline *vs*. 12^th^ month, for baseline *vs*. 18^th^ month in CKD stage 4+RSD group; eGFR (CKD-EPI): ^†^P<0.05 for baseline *vs*. 12^th^ month, for baseline *vs*. 18^th^ month in CKD stage 3 group, ^†^P<0.05 for baseline *vs*. 18^th^ month, for 12^th^ *vs*. 18^th^ month in CKD stage 4 group, and ^††^P<0.0001 for baseline *vs*. 12^th^ month, for baseline *vs*. 18^th^ month, for 12^th^ *vs*. 18^th^ month in CKD stage 4+RSD group.

### Echocardiographic parameters

At baseline, there were significant differences between CKD stages groups related to LV mass index for all the comparisons except for CKD stage 4 vs. 4+RSD, being watched significant differences for all the comparisons at 18 months of follow-up. At the baseline, comparisons between groups regarding LV ejection fraction, end diastolic LV internal dimension and end systolic LV internal dimension did not show the difference. However, at the 18^th^ month of follow-up, we observed significant differences concerning CKD stages for LV ejection fraction in the following comparisons between CKD stages: 1 and 3, 2 and 3, 1 and 4, 1 and 4+RSD, 2 and 4, 2 and 4+RSD, 3 and 4+RSD, 4 and 4+RSD. At the 18^th^ month of follow-up, changes were also observed for end diastolic LV internal dimension only in the following comparisons between CKD stages: 1 and 3, 1 and 4, as well 4 and 4+RSD. Into the same group, comparisons between values at baseline and the 18^th^ month showed a significant difference for LV mass index in all the CKD groups, and for LV ejection fraction this difference was only noted in patients with CKD stage 3, 4 and 4+RSD (Table 3).

**Table 3:** Echocardiographic parameters during 18 months of follow-up

Baseline	CKD stage 1 (n=25)	CKD stage 2 (n=25)	CKD stage 3 (n=25)	CKD stage 4 (n=20)	CKD stage 4+RSD (n=20)	Overall P value for comparisons between groups
LVMI, g/m^2^	102.3±10.0^††^	117.4±9.5^††^	139.1±9.8^††^	155.7±11.1^††^	153.4±7.8^▲††^	<0.0001 for all comparisons except for CKD stage 4 *vs*. 4+RSD
LVEF, %	30.7±4.8	29.2±5.5	29.5±5.0^††^	28.4±6.0^†^	27.8±5.3^†^	0.9812
LVIDED, mm	60.1±12.2	61.6±10.8	67.0±14.7	68.3±14.5	67.3±11.4	0.1032
LVIDES, mm	51.0±16.2	52.3±14.4	53.1±16.5	54.0±18.0	53.5±15.6	0.9743
12^th^ month of follow-up	CKD stage 1 (n=25)	CKD stage 2 (n=25)	CKD stage 3 (n=25)	CKD stage 4 (n=20)	CKD stage 4+RSD (n=20)	Overall P value for comparisons between groups
LVMI, g/m^2^	113.1±10.5	127.5±9.0	150.4±8.5^†^	175.2±14.0^†^	112.5±12.2^▲†^	<0.0001 for all comparisons except for CKD stage 1 *vs*. 4+RSD
LVEF, %	31.3±5.3	29.0±4.8	26.8±3.9	24.9±4.3	31.8±2.0	<0.0001^*/*^*
LVIDED, mm	58.7±14.2	62.0±10.0	66.2±16.1	68.5±10.0	62.0±10.4	0.0805
LVIDES, mm	52.0±15.5	53.0±13.8	54.0±16.3	55.1±16.9	54.0±15.1	0.9713
18^th^ month of follow-up	CKD stage 1 (n=25)	CKD stage 2 (n=25)	CKD stage 3 (n=25)	CKD stage 4 (n=20)	CKD stage 4+RSD (n=20)	Overall P value for comparisons between groups
LVMI, g/m^2^	118.0±9.5	132.2±9.3	157.1±9.9	189.3±11.2	101.5±9.3	<0.0001 for all comparisons
LVEF, %	30.5±4.5	29.5±5.0	25.0±3.2	22.5±3.8^††^	36.5±2.7^††^	<0.0001^*/*^*
LVIDED, mm	59.5±9.6	62.4±10.1	67.2±9.8	70.2±8.7	60.1±9.0	0.0007*
LVIDES, mm	52.5±8.8	53.2±9.6	56.0±10.0	57.8±8.0	52.1±9.6	0.1984

The values are presented as mean ± SD. CKD, chronic kidney disease (stage 1, 2, 3 and 4); LVMI, left ventricular mass index; LVEF, left ventricular ejection fraction measured by Simpson's method; LVIDED, left ventricle internal dimension at the end of diastole; LVIDES, left ventricle internal dimension at the end of systole; +RSD, patients that underwent renal sympathetic denervation. Comparisons between groups, LVMI: ^▲^P=0.9442 for CKD stage 4 *vs*. 4+RSD and P=0.9997 for CKD stage 1 *vs*. 4+RSD; LVIDED: at 18^th^ month of follow-up *P<0.05 for CKD stage 1 *vs*. 3, for CKD stage 1 *vs*. 4, for CKD stage 4 *vs*. 4+RSD; LVEF: at 12^th^ month of follow-up *P<0.05 for CKD stage 1 *vs*. 3, for CKD stage 2 *vs*. 4, for CKD stage 3 *vs*. 4, and ^*^P<0.0001 for CKD stage 1 *vs*. 4, for CKD stage 4 *vs*. 4+RSD, at 18^th^ month of follow-up *P<0.05 for CKD stage 2 *vs*. 3, and ^*^P<0.0001 for CKD stage 1 *vs*. 3, for CKD stage 1 *vs*. 4, for CKD stage 1 *vs*. 4+RSD, for CKD stage 2 *vs*. 4, for CKD stage 2 *vs*. 4+RSD, for CKD stage 3 *vs*. 4+RSD, for CKD stage 4 *vs*. 4+RSD. Comparisons into the same group, LVMI: ^††^P<0.0001 for baseline *vs*. 12^th^ month, for baseline *vs*. 18^th^ month in CKD stage 1 and 2 groups; ^†^P<0.05 for 12^th^ *vs*. 18^th^ month, ^††^P<0.0001 for baseline *vs*. 12^th^ month, for baseline *vs*. 18^th^ month in CKD stage 3, 4 and 4+RSD groups; LVEF: ^††^P<0.0001 for baseline *vs*. 18^th^ month in CKD stage 3 group; ^†^P<0.05 for baseline *vs*. 12^th^ month, ^††^P<0.0001 for baseline *vs*. 18^th^ month and for 12^th^ *vs*. 18^th^ month in CKD stage 4 and 4+RSD groups.

### Stimulation of the nerves of the renal arteries

There was a significant association concerning the difference (Δ) in invasive systolic BP and VT events for each quarter of the both renal arteries, evaluated by Pearson technique [5], while RNS was performed for individuals on stage 4 of CKD that would undergo RNS or not (Table 4). According to this table, some zones are furthermost prone to rise of the invasive systolic BP concurrently with VT manifestation in these patients. Twenty individuals on stage 4 of CKD who have not been submitted to RSD demonstrated area under the roc curve (AUC)= 0.9993/0.9985, 95% confidence interval (CI)=0.9965 - 0.9997/0.9958 - 0.9994, P <0.0001/<0.0001 [5], the limit point of Δ invasive systolic BP to generate VT in the course of RNS was higher than 25.5 mmHg/25.5 mmHg, and sensitivity= 95%/98%, specificity= 99%/100%, for the left renal artery and right renal artery, respectively. The further twenty subjects on stage 4 of CKD who were submitted to RSD successively showed the AUC= 0.9980/0.9990, 95% CI=0.9956 - 0.9993/0.9986 - 0.9995, P <0.0001/<0.0001, sensitivity= 96%/99%, specificity= 100%/100%, and the limit point of Δ invasive systolic BP to initiate VT in the course of RNS was higher than 25.5 mmHg/24.5 mmHg, for left and right renal arteries, correspondingly [5].

**Table 4:** Data of the renal nerve stimulation in CKD patients on stage 4 (n=40 patients)

Sites	20 patients = 40 left renal arteries (n=640 sites)				20 patients = 40 right renal arteries (n=640 sites)			
RNS per quadrant	Sites where VT occurred during RNS, n (%)		Δ mean invasive systolic BP during RNS, mmHg		Sites where VT occurred during RNS, n (%)		Δ mean invasive systolic BP during RNS, mmHg	
	RNSgroup20 LRA(n=320)	RNS+RSD group20 LRA(n=320)	RNSgroup20 LRA(n=320)	RNS+RSD group20 LRA(n=320)	RNSgroup20 RRA(n=320)	RNS+RSD group20 RRA(n=320)	RNSgroup20 RRA(n=320)	RNS+RSD group20 RRA(n=320)
Quadrant1 - Ostium	14 (70%)	12 (60%)	27.6	26.3	13 (65%)	18 (90%)	27.3	29.5
Quadrant2 - Ostium	14 (70%)	14 (70%)	28.4	27.4	9 (45%)	18 (90%)*	26.8	30.2
Quadrant3 - Ostium	11 (55%)	15 (75%)	26.3	28.4	9 (45%)	19 (95%)^*^	25.6	29.6*
Quadrant4 - Ostium	15 (75%)	17 (85%)	27.8	30.4*	0 (0%)	0 (0%)	6.8	6.5^**^*
Quadrant1 - Proximal	17 (85%)	20 (100%)	27.8	29.7	10 (50%)	0 (0%)^*^	28.9	8.2
Quadrant2 - Proximal	12 (60%)	0 (0%)^**^*	25.7	10.0^**^*	12 (60%)	20 (100%)*	28.8	31.5
Quadrant3 - Proximal	0 (0%)	0 (0%)	8.3	10.8	0 (0%)	0 (0%)	6.0	4.3
Quadrant4 - Proximal	0 (0%)	0 (0%)	7.3	9.2	0 (0%)	0 (0%)	6.9	7.7
Quadrant1 - Middle	0 (0%)	0 (0%)	5.8	8.7	0 (0%)	0 (0%)	7.5	6.3
Quadrant2 - Middle	0 (0%)	0 (0%)	7.1	7.1	0 (0%)	0 (0%)	5.8	4.8
Quadrant3 - Middle	15 (75%)	20 (100%)*	28.2	30.4	9 (45%)	20 (100%)^**^*	25.1	30.3^**^*
Quadrant4 - Middle	0 (0%)	0 (0%)	4.2	3.0	0 (0%)	0 (0%)	3.6	6.0
Quadrant1 - Distal	0 (0%)	0 (0%)	4.9	4.6	12 (60%)	15 (75%)	30.3	28.7
Quadrant2 - Distal	0 (0%)	20 (100%)^**^*	7.9	30.9^**^*	14 (70%)	0 (0%)^**^*	29.8	7.5^**^*
Quadrant3 - Distal	14 (70%)	14 (70%)	29.6	28.3	0 (0%)	0 (0%)	6.3	5.4
Quadrant4 - Distal	12 (60%%)	0 (0%)^**^*	28.9	6.8^**^*	0 (0%)	16 (80%)^**^*	6.4	28.8^**^*
Pearson correlation; 95% CI; P value	RNS group: r=0.9809; 95%CI: 0.9445–0.9935; P<0.0001				RNS group: r=0.9825; 95%CI: 0.9489–0.9941; P<0.0001			
Pearson correlation; 95% CI; P value	RNS+RSD group: r=0.9726; 95%CI: 0.9209–0.9907; P<0.0001				RNS+RSD group: r=0.9925; 95%CI: 0.9780–0.9455; P<0.0001			

BP, blood pressure; CKD, chronic kidney disease; Δ, variation; LRA, left renal artery; RNS, renal nerve stimulation; RRA, right renal artery; RSD, renal sympathetic denervation; VT, ventricular tachycardia; 95%CI, 95% Confidence Interval;*P<0.05, ^*^P<0.001, and ^**^*P<0.0001 for comparisons between RNS *vs*. RNS+RSD groups during RNS of renal arteries at the same side of the body.

### Therapy events

**Figure 1:**
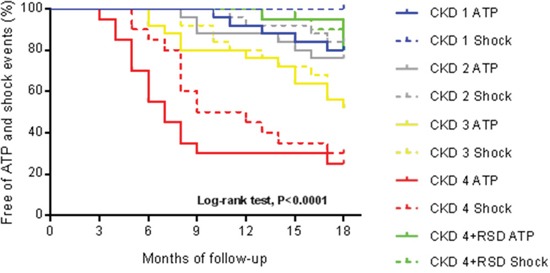
The Kaplan-Meier curves describe event occurrences of anti-tachycardia pacing therapy (ATP) and shock at different stages of chronic kidney disease (CKD), during 18 months CKD stage 1, N=25; CKD stage 2, N=25; CKD stage 3, N=25; CKD stage 4, N=20; CKD stage 4+RSD, N=20. +RSD, patients that underwent renal sympathetic denervation.

Table 5 shows the number of ATP and shock events recorded by ICD during 1 year and a half of monitoring, and quantity differences in therapeutic events between the various stages of CKD. The ATP and shock events between all the CKD stages was assessed using the the log-rank/Mantel-Haenszel test; then we could get values of hazard ratio (HR), 95% CI and P, as shown in Table 6. After 18 months of monitoring, 75% of the individuals with CKD stage 4 received ATP, and 70% were treated with shock while only 20% of the subjects with CKD stage 4 that were submitted to RSD received ATP, and 20% were treated with shock, P<0.0001 and P=0.0002, respectively, by log-rank/Mantel-Haenszel test (Figure 1).

**Table 5:** ATP and shock events recorded by automatic cardioverter defibrillator during follow-up

Number of events	CKD stage 1 (n=25)	CKD stage 2 (n=25)	CKD stage 3 (n=25)	CKD stage 4 (n=20)	CKD stage 4+RSD (n=20)	Overall P value for comparisons between groups
18 months of follow-up						
ATP	8.8±2.0	13.6±1.5	27.4±2.2	48.8±4.6	10.0±3.2^▲^	<0.0001 for all comparisons except for CKD stage 1 *vs*. 4+RSD
Shocks	0	3.8±0.9	13.1±2.4	38.3±2.1	1.5±0.9*	<0.0001 for all comparisons except for CKD stage 1 *vs*. 4+RSD

The values are presented as mean ± SD. ATP, anti-tachycardia pacing therapy; CKD, chronic kidney disease; +RSD, patients that underwent renal sympathetic denervation. ^▲^P=0.6109 for CKD stage 1 *vs*. 4+RSD; *P=0.0122 for CKD stage 1 *vs*. 4+RSD.

**Table 6:** Hazard ratio for ATP and shock events between the CKD stages, evaluated by log-rank/Mantel-Haenszel tests

ATP events	18^th^ month of follow-up		
	Hazard Ratio	95% Confidence Interval	P value
CKD stage 2 *vs*. 1	1.265	0.388 – 4.127	0.6957
CKD stage 3 *vs*. 1	2.862	1.102 – 7.432	0.0362
CKD stage 4 *vs*. 1	8.939	3.359 – 23.790	<0.0001
CKD stage 4+RSD *vs*. 1	0.903	0.244 – 3.341	0.8769
CKD stage 3 *vs*. 2	2.305	0.913 – 5.819	0.0813
CKD stage 4 *vs*. 2	7.341	2.827 – 19.060	<0.0001
CKD stage 4+RSD *vs*. 2	0.735	0.213 – 2.544	0.6286
CKD stage 4 *vs*. 3	3.146	1.358 – 7.286	0.0075
CKD stage 4+RSD *vs*. 3	0.314	0.118 – 0.839	0.0303
CKD stage 4+RSD *vs*. 4	0.127	0.047 – 0.338	<0.0001
Shock events			
CKD stage 2 *vs*. 1	7.895	1.110 – 56.130	0.0390
CKD stage 3 *vs*. 1	10.300	3.246 – 32.710	<0.0001
CKD stage 4 *vs*. 1	20.000	6.481 – 61.690	<0.0001
CKD stage 4+RSD *vs*. 1	10.630	1.449 – 77.960	0.0201
CKD stage 3 *vs*. 2	3.607	1.348 – 9.651	0.0156
CKD stage 4 *vs*. 2	8.073	2.956 – 22.050	<0.0001
CKD stage 4+RSD *vs*. 2	1.197	0.298 – 4.812	0.7981
CKD stage 4 *vs*. 3	2.522	1.089 – 5.840	0.0309
CKD stage 4+RSD *vs*. 3	0.325	0.122 – 0.865	0.0365
CKD stage 4+RSD *vs*. 4	0.156	0.058 – 0.419	0.0002

ATP, anti-tachycardia therapy pacing; CKD, chronic kidney disease; +RSD, patients that underwent renal sympathetic denervation.

## DISCUSSION

In the present study, the RSD reduced the greater occurrence of arrhythmic events, appropriate ICD therapies in the CKD later phases, as well as, the greater risk rates for these events in CKD patients on stage 4 reported previously by our group [15]. Our results suggest that the RSD can be a powerful tool to reduce the rate of lethal arrhythmic events in patients with CKD later phases.

The hypertensive and diabetic rate of patients in all stages of CKD did not differ. So, as mentioned previously, it can be speculated that sympathetic overactivity of CKD contributes from the premature clinical step of the illness, displaying a direct correlation with the gravity of the end stage of the renal disease [20–23]. Such reduction in the glomerular filtration rate happens, augmenting the compatibly in cardiovascular incidents and death in CKD individuals [5, 24], principally because of arrhythmic manifestations and their concerns. The renal impairment prompts restructuring of the heart, comprising left ventricular hypertrophy (LVH), as well as, fibrosis of the heart, showing an autonomous link between chronic renal impairment and LVH [25–28]. Precisely, may be noted a gradual growth in the LVH occurrence, and augmented LV mass while the glomerular filtration rate drops. Furthermore, surrounded by individuals with end stage of renal disease in hemodialysis, magnetic resonance imaging exhibits a diffuse pattern form with gadolinium uptake, indicative of no ischemic cardiomyopathy and necrosis [20]. The pathogenesis of these conditions is considered multifactorial [29–31]. Moreover, CKD is correlated with vascular problems, comprising inurement of the vessels by calcium buildup [32–35]. The decrease in glomerular filtration rate and endothelial dysfunction are interconnected developments leading to a reduction of vessels stretch and afterwards intensification of ischemia manifestations. Researches involving humans have shown reduced vasodilation, which is reliant on endothelium and is linked to minor kidney insufficiency [36, 37]. If not well managed, such disorders make headway self-reliantly and create a recurring association resulting in damage to the kidneys and the vessels. Subsequently, restructuring and vascular sclerosis may pledge the perfusion backup, as well as, surge the chances of ischemia [38] that function as mutual activating elements to start arrhythmic events. Further, organizational variations maybe induce modifications in the myocardial electrophysiological features. The necrosis of the myocardium disorders the standard structure generating a reduction in the transmission speed across the unstructured fibers [39]. Such disorder usually leads to the formation of non-homogeneous zones of transmission and depolarization, able to maintain a reentrant arrhythmic event, as sustained VT for example [32, 40]. These organizational alterations in cardiac impulse transmission delay the stimulation of the ventricles besides generate a tardy potential in the end fragment of the QRS. In the dependent reentrant arrhythmias scars, dissimilar zones forming electrical conveyance, kidneys failure, besides to surge the danger of spontaneous arrhythmia or activated by further activate points [41].

Recently, Hering and colleagues reported that the RSD in hypertensive resistant subjects provoked an important decrease in BP allied to a considerable and fast decrease in the singular shooting characteristics of sympathetic vasoconstrictor filaments, using the method of muscle sympathetic nerve activity (MSNA), matching refractory hypertensive subjects who not underwent the procedure, post 3 months of monitoring [42].

Remo and colleagues related a sequence of cases that gave us hopeful initial information on the security and efficacy of RSD as an accessorial tool to treat subjects bearing cardiomyopathy and VT refractory to usual ablations [43], as well as, Armaganijan and colleagues described the significance of sympathetic triggering for subjects presenting VT and proposed a possible place for RSD to decrease the burden of ventricular arrhythmias [44].

So we can conclude that some modifications that occur in patients with CKD leading to malignant ventricular arrhythmias can be modified by RSD. Our results suggest that RSD is effective in more advanced CKD stages, decreasing the incidence of these arrhythmias hence the number of ATP and shock events.

### Limitations

Although our data show a greater incidence of arrhythmias and hence therapies in advanced stages of CKD, our group of patients was small. This relatively small sample size can be seen as a limitation. In future studies, the MSNA should be assessed, contributing importantly to evaluate the level of sympathetic interruption.

## MATERIALS AND METHODS

### Study design

This prospective study was piloted at the Department of Cardiac Artificial Stimulation and Cardiac Surgery of the Hospital e Clínica São Gonçalo, São Gonçalo, Rio de Janeiro, Brazil in partnership with Elisabethinen Krankenhaus, Linz, Austria. A cohort of patients received standard therapy for primary or secondary prevention of sudden cardiac death (SCD) in patients with structural heart disease, subjected to the ICD-DR implant according to the “Guidelines for Implantable Electronic Cardiac Devices of the Brazilian Society of Cardiology” [16].

Patients were followed for one year and a half after the implant procedure. Inclusion criteria were as follows: (i) subjects with structural heart disease and ICD implantation indication for primary or secondary prevention of SCD; (ii) left ventricular ejection fraction ≤35%; (iii) patients who provided documentation not presenting cardiac ischemia before ICD implantation evidenced by myocardial scintigraphy at rest and during stress, by cardiac magnetic resonance imaging at rest and during stress, or coronary angiography; (iv) estimated glomerular filtration rate (eGFR) by the CKD-EPI (Chronic Kidney Disease Epidemiology Collaboration) equation, eGFR^17^ >15 mL/min/1.73m^2^ (patients presenting eGFR >60 mL/min/1.73m^2^ were obliged to present microalbuminuria).

Exclusion criteria were: (i) ischemic heart disease; (ii) LVEF> 35%; (iii) absence of structural heart disease; (iv) valvar heart disease that might lead to arrhythmias; (v) the presence of previously documented atrial fibrillation.

The recruitment of the patients began in January 2012 and ended in June 2015. We enrolled 115 patients with CKD, being 25 on stage 1, 25 on stage 2, 25 on stage 3, and 40 on stage 4. They were followed up for 18 months after ICD implantation, and they were identified in our offices. The study was conducted in agreement with the Declaration of Helsinki and was approved by the Ethics Committee of our hospital. All individuals provided written informed consent before inclusion in the study.

The 40 subjects with CKD on stage 4 were randomly divided into two groups (RNS, n=20, and RNS+RSD, n=20). All of them were followed for exactly 18 months to assess all the parameters measured in this investigation. This study was double-blind, and neither the patient nor the clinician responsible for follow-up of the ICD and other parameter assessments was aware of whether RSD had been performed; only the physician operator had this information.

### Implantation and programming of the ICDs, twenty-four hour ABPM, and transthoracic echocardiography

These procedures were previously reported in detail in our previously published manuscript [15].

### Follow-up patients

The patients were evaluated 15 days after ICD implantation to observe the pocket, the site of the surgical incision, and to adjust the device programming. Fifteen days later, the patients returned for further evaluation (one month after ICD implantation). The data were obtained from the day of the device implant to 18 months after implantation. Subsequently, patients were evaluated every 3 months till the complete total period of follow-up. At each follow-up visit, we achieved a record (stored on a USB device and then transferred to a computer) of the ICD memory data that has accumulated since the last reset of memory. The occurrence and duration of ATP and shock events were recorded.

### Renal nerve stimulation

The 80 (40 leftt and 40 right) renal arteries from the 40 CKD patients on stage 4 were stimulated according to 16 pattern quadrant previously described by our group [18]. After the stimulation, we waited for the BP to return to baseline values and when ventricular tachycardia (VT) event occurred together, we also waited for the rhythm return to the sinus rhythm, what happened spontaneously after stopping the RNS, and before proceeding to the next stimulation site. The patients remained hospitalized in the ward for 24 h after the procedure.

### Renal sympathetic denervation

Twenty CKD patients on stage 4 underwent RSD guided by RNS at baseline and were followed until the 18^th^ month post procedure. The RSD was previously described in detail by our group [19].

### Statistical analysis

All patients enrolled were included in the analysis. The results were expressed as the mean and standard deviation (mean ± SD) in the case of normal distribution and as median with interquartile range otherwise. Statistical tests were all of two sides. Comparisons between the two paired values were performed by paired t-test in case of a Gaussian distribution or alternatively, by Wilcoxon test. The comparisons between more than two values paired values were performed by analysis of variance for repeated measures ANOVA or Kruskal-Wallis test, as appropriate, complemented by a post hoc test. Frequencies were compared with *x*^2^ or Fisher's exact tests. P values <0.05 were considered significant. Correlations between two variables were performed by Pearson in the case of a Gaussian distribution or, alternatively, with the Spearman correlation test. Kaplan-Meier analysis was performed to determine the probability of success, assessed as the percentage of patients free of therapies. Differences in free survival therapies were evaluated with the log-rank/Mantel-Haenszel test. The Cox regression analysis was applied to explore triggering factors of ATP and shock events. All statistical analyzes were performed using the program Graphpad Prism v 7.0 (Graphpad software, La Jolla, CA, USA).

## CONCLUSION

Our results show a decay in the rate of ventricular arrhythmic events, as well as, appropriate ICD therapies in advanced stages of CKD, reducing the risk rates for these events in patients on stage 4 of CKD after RSD steered by RNS in contrast with the other CKD stages. Such data propose that RSD is able to manage the greater occurrence of lethal arrhythmic events in CKD later phases.

## Abbreviations

ABPM: ambulatory blood pressure measurements.
ATP: Anti-tachycardia Therapy Pacing.
AUC: area under the roc curve.
BP: blood pressure.
CI: confidence interval.
CKD: chronic kidney disease.
eGFR: estimated glomerular filtration rate.
HR: hazard ratio.
ICD: automatic implantable cardioverter-defibrillator.
LV: left ventricular.
LVEF: left ventricular ejection fraction.
LVH: left ventricular hypertrophy.
MSNA: muscle sympathetic nerve activity.
RNS: renal nerve stimulation.
RSD: renal sympathetic denervation.
SCD: sudden cardiac death.
VT: ventricular tachycardia.

## References

[1] Roger VL, Go AS, Lloyd-Jones DM, Benjamin EJ, Berry JD, Borden WB, Bravata DM, Dai S, Ford ES, Fox CS, Fullerton HJ, Gillespie C, Hailpern SM (2012). Heart disease and stroke statistics—2012 update: a report from the American Heart Association. Circulation.

[2] Myerburg RJ, Junttila MJ (2012). Sudden cardiac death caused by coronary heart disease. Circulation.

[3] Moss AJ, Hall WJ, Cannom DS, Daubert JP, Higgins SL, Klein H, Levine JH, Saksena S, Waldo AL, Wilber D, Brown MW, Heo M (1996). Improved survival with an implanted defibrillator in patients with coronary disease at high risk for ventricular arrhythmia. Multicenter Automatic Defibrillator Implantation Trial Investigators. N Engl J Med.

[4] Moss AJ, Zareba W, Hall WJ, Klein H, Wilber DJ, Cannom DS, Daubert JP, Higgins SL, Brown MW, Andrews ML (2002). Multicenter Automatic Defibrillator Implantation Trial II Investigators. Prophylactic implantation of a defibrillator in patients with myocardial infarction and reduced ejection fraction. N Engl J Med.

[5] Kiuchi MG, Chen S (2017). Acute effects of renal sympathetic denervation guided by renal nerve stimulation in CKD patients with ICD. J Integr Cardiol.

[6] Goldstein S, Landis JR, Leighton R, Ritter G, Vasu CM, Wolfe RA, Acheson A, VanderBrug Medendorp S (1985). Predictive survival models for resuscitated victims of out-of-hospital cardiac arrest with coronary heart disease. Circulation.

[7] Grassi G (2010). Sympathetic neural activity in hypertension and related diseases. Am J Hypertens.

[8] Grassi G (2009). Assessment of sympathetic cardiovascular drive in human hypertension: achievements and perspectives. Hypertension.

[9] Paton JF, Raizada MK (2010). Neurogenic hypertension. Exp Physiol.

[10] Tinucci T, Abrahao SB, Santello JL, Mion D (2001). Mild chronic renal insufficiency induces sympathetic overactivity. J Hum Hypertens.

[11] Schlaich MP, Socratous F, Hennebry S, Eikelis N, Lambert EA, Straznicky N, Esler MD, Lambert GW (2009). Sympathetic activation in chronic renal failure. J Am Soc Nephrol.

[12] Neumann J, Ligtenberg G, Klein II, Koomans HA, Blankestijn PJ (2004). Sympathetic hyperactivity in chronic kidney disease: pathogenesis, clinical relevance, and treatment. Kidney Int.

[13] Grassi G, Bertoli S, Seravalle G (2012). Sympathetic nervous system: role in hypertension and in chronic kidney disease. Curr Opin Nephrol Hypertens.

[14] Go AS, Chertow GM, Fan D, McCulloch CE, Hsu CY (2004). Chronic kidney disease and the risks of death, cardiovascular events, and hospitalization. N Engl J Med.

[15] Kiuchi MG, Chen S, Pürerfellner H (2017). Incidence of ventricular arrhythmic events in CKD patients with ICD. Int J Cardiol.

[16] Martinelli Filho M, Zimerman LI, Lorga AM, Vasconcelos JTM, Rassi A (2007). Guidelines for Implantable Electronic Cardiac Devices of the Brazilian Society of Cardiology. Arq Bras Cardiol.

[17] Levey AS, Stevens LA, Schmid CH, Zhang YL, Castro AF, Feldman HI, Kusek JW, Eggers P, Van Lente F, Greene T, Coresh J, CKD-EPI (Chronic Kidney Disease Epidemiology Collaboration) (2009). A new equation to estimate glomerular filtration rate. Ann Intern Med.

[18] Kiuchi MG, Chen S (2016). Renal sympathetic stimulation in patients with controlled hypertension and paroxysmal atrial fibrillation. Int J Cardiol.

[19] Kiuchi MG, Mion D, Graciano ML, MA de Queiroz Carreira, Kiuchi T, Chen S, Lugon JR (2016). Proof of concept study: Improvement of echocardiographic parameters after renal sympathetic denervation in CKD refractory hypertensive patients. Int J Cardiol.

[20] Sarnak MJ, Katz R, Stehman-Breen CO, Fried LF, Jenny NS, Psaty BM, Newman AB, Siscovick D, Shlipak MG, Cardiovascular Health Study (2005). Cystatin C concentration as a risk factor for heart failure in older adults. Ann Intern Med.

[21] Deo R, Fyr CL, Fried LF, Newman AB, Harris TB, Angleman S, Green C, Kritchevsky SB, Chertow GM, Cummings SR, Shlipak MG, Health ABC study (2008). Kidney dysfunction and fatal cardiovascular disease—an association independent of atherosclerotic events: results from the Health, Aging, and Body Composition (Health ABC) study. Am Heart J.

[22] Kannel WB, Cupples LA, D'Agostino RB (1987). Sudden death risk in overt coronary heart disease: the Framingham Study. Am Heart J.

[23] Stevenson WG, Stevenson LW, Middlekauff HR, Saxon LA (1993). Sudden death prevention in patients with advanced ventricular dysfunction. Circulation.

[24] Epstein AE, DiMarco JP, Ellenbogen KA, Estes NA, Freedman RA, Gettes LS, Gillinov AM, Gregoratos G, Hammill SC, Hayes DL, Hlatky MA, Newby LK, Page RL (2008). ACC/AHA/HRS 2008 Guidelines for Device-Based Therapy of Cardiac Rhythm Abnormalitiesa report of the American College of Cardiology/American Heart Association Task Force on Practice Guidelines (Writing Committee to Revise the ACC/AHA/NASPE 2002 Guideline Update for Implantation of Cardiac Pacemakers and AntiarrhythmiaDevices): developed in collaboration with the American Association for Thoracic Surgery and Society of Thoracic Surgeons. Circulation.

[25] Cerasola G, Nardi E, Mulè G, Palermo A, Cusimano P, Guarneri M, Arsena R, Giammarresi G, Carola Foraci A, Cottone S (2010). Left ventricular mass in hypertensive patients with mild-to-moderate reduction of renal function. Nephrology (Carlton).

[26] Levin A, Thompson CR, Ethier J, Carlisle EJ, Tobe S, Mendelssohn D, Burgess E, Jindal K, Barrett B, Singer J, Djurdjev O (1999). Left ventricular mass index increase in early renal disease: impact of the decline in hemoglobin. Am J Kidney Dis.

[27] Paoletti E, Bellino D, Cassottana P, Rolla D, Cannella G (2005). Left ventricular hypertrophy in nondiabetic predialysis CKD. Am J Kidney Dis.

[28] Moran A, Katz R, Jenny NS, Astor B, Bluemke DA, Lima JA, Siscovick D, Bertoni AG, Shlipak MG (2008). Left ventricular hypertrophy in mild and moderate reduction in kidney function determined using cardiac magnetic resonance imaging and cystatin C: the multi-ethnic study of atherosclerosis (MESA). Am J Kidney Dis.

[29] Cioffi G, Tarantini L, Frizzi R, Stefenelli C, Russo TE, Selmi A, Toller C, Furlanello F, de Simone G (2011). Chronic kidney disease elicits excessive increase in left ventricular mass growth in patients at increased risk for cardiovascular events. J Hypertens.

[30] Schroeder AP, Kristensen BO, Nielsen CB, Pedersen EB (1997). Heart function in patients with chronic glomerulonephritis and mildly to moderately impaired renal function. An echocardiographic study. Blood Press.

[31] Hunter JJ, Chien KR (1999). Signaling pathways for cardiac hypertrophy and failure. N Engl J Med.

[32] Pai AS, Giachelli CM (2010). Matrix remodeling in vascular calcification associated with chronic kidney disease. J Am Soc Nephrol.

[33] Briet M, Collin C, Karras A, Laurent S, Bozec E, Jacquot C, Stengel B, Houillier P, Froissart M, Boutouyrie P, Nephrotest Study Group (2011). Arterial remodeling associates with CKD progression. J Am Soc Nephrol.

[34] Hu MC, Shi M, Zhang J, Quiñones H, Griffith C, Kuro-o M, Moe OW (2011). Klotho deficiency causes vascular calcification in chronic kidney disease. J Am Soc Nephrol.

[35] Shroff R, Shanahan CM (2011). Klotho: an elixir of youth for the vasculature?. J Am Soc Nephrol.

[36] Perticone F, Maio R, Tripepi G, Zoccali C (2004). Endothelial dysfunction and mild renal insufficiency in essential hypertension. Circulation.

[37] Perticone F, Maio R, Perticone M, Sciacqua A, Shehaj E, Naccarato P, Sesti G (2010). Endothelial dysfunction and subsequent decline in glomerular filtration rate in hypertensive patients. Circulation.

[38] Kingwell BA, Waddell TK, Medley TL, Cameron JD, Dart AM (2002). Large artery stiffness predicts ischemic threshold in patients with coronary artery disease. J Am Coll Cardiol.

[39] Waldo AL, Plumb VJ, Arciniegas JG, MacLean WA, Cooper TB, Priest MF, James TN (1983). Transient entrainment and interruption of the atrioventricular bypass pathway type of paroxysmal atrial tachycardia. A model for understanding and identifying reentrant arrhythmias. Circulation.

[40] Schmidt A, Azevedo CF, Cheng A, Gupta SN, Bluemke DA, Foo TK, Gerstenblith G, Weiss RG, Marbán E, Tomaselli GF, Lima JA, Wu KC (2007). Infarct tissue heterogeneity by magnetic resonance imaging identifies enhanced cardiac arrhythmia susceptibility in patients with left ventricular dysfunction. Circulation.

[41] Brotman DJ, Bash LD, Qayyum R, Crews D, Whitsel EA, Astor BC, Coresh J (2010). Heart rate variability predicts ESRD and CKD-related hospitalization. J Am Soc Nephrol.

[42] Hering D, Lambert EA, Marusic P, Walton AS, Krum H, Lambert GW, Esler MD, Schlaich MP (2013). Substantial reduction in single sympathetic nerve firing after renal denervation in patients with resistanthypertension. Hypertension.

[43] Remo BF, Preminger M, Bradfield J, Mittal S, Boyle N, Gupta A, Shivkumar K, Steinberg JS, Dickfeld T (2014). Safety and efficacy of renal denervation as a novel treatment of ventricular tachycardia storm in patients with cardiomyopathy. Heart Rhythm.

[44] Armaganijan LV, Staico R, Moreira DA, Lopes RD, Medeiros PT, Habib R, Melo Neto J, Katz M, Armaganijan D, Sousa AG, Mahfoud F, Abizaid A (2015). 6-month outcomes in patients with implantable cardioverter-defibrillators undergoing renal sympathetic denervation for the treatment of refractory ventricular arrhythmias. JACC Cardiovasc Interv.

